# Nucleolin and nucleophosmin expression patterns in pulmonary adenocarcinoma invading the pleura and in pleural malignant mesothelioma

**DOI:** 10.1111/1759-7714.13564

**Published:** 2020-07-16

**Authors:** Marek Masiuk, Piotr Waloszczyk, Magdalena Lewandowska, Ewa Dobak, Elzbieta Urasinska

**Affiliations:** ^1^ Department of Pathology Pomeranian Medical University Szczecin Poland; ^2^ Independent Laboratory of Pathology “Zdunomed” LLC Szczecin Poland

**Keywords:** Adenocarcinoma, mesothelioma, nucleolin, nucleophosmin, pleura

## Abstract

**Background:**

Visceral pleural invasion (VPI) in adenocarcinoma of the lung is considered a poor prognostic factor. The purpose of this study was to analyze nucleolin and nucleophosmin expression in pulmonary adenocarcinoma (PA) with VPI and in pleural malignant mesothelioma.

**Methods:**

The study was conducted on the basis of 19 pathologically‐confirmed cases of adenocarcinoma of the lung and 29 cases of epithelioid malignant mesothelioma. The nucleolin and nucleophosmin expression was assessed immunohistochemically and analyzed with image analysis software.

**Results:**

Nucleolin expression was lower while nucleophosmin was higher in pleural invasion of pulmonary adenocarcinoma than in the central part of the tumor. Differences in subpopulations of cells with different expression of proteins studied were also found. Malignant mesothelioma showed lower nucleolin expression than adenocarcinoma of the lung but no differences in nucleophosmin expression were found.

**Conclusions:**

The results of our study suggested that lower nucleolin and higher nucleophosmin expression may be related to higher invasiveness of adenocarcinoma of the lung. Differences in nucleolin expression between pulmonary adenocarcinoma and malignant mesothelioma indicate another aspect of biology of these pleura‐invading cancers that requires further study.

**Key points:**

**Significant findings of the study:**

Differences in nucleolin and nucleophosmin expression in pleura invading pulmonary adenocarcinoma indicate the involvement of these proteins in its locoregional spread while differences in nucleolin expression between pulmonary adenocarcinoma and malignant mesothelioma suggest another aspect of biology of these cancers.

**What this study adds:**

This is the first study on nucleolin and nucleophosmin expression in pleural malignant mesothelioma and pleura‐invading pulmonary adenocarcinoma. Our findings may assist in understanding the mechanisms of locoregional spread of adenocarcinoma and differences between these two pleura‐invading cancers.

## Graphical abstract

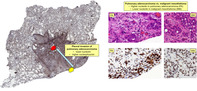

The lower nucleolin expression and higher nucleophosmin expression were related to the pleural (locoregional) invasion of pulmonary adenocarcinoma. Pleural malignant mesothelioma showed lower nucleolin expression than pleura invading pulmonary adenocarcinoma

## Introduction

Lung cancer, especially non‐small cell lung cancer (NSCLC), is one of the most common cancers worldwide and the main cause of cancer death. Among different histologic types of lung cancer adenocarcinoma is the most common and its incidence is increasing. It typically localizes at the periphery of the lung and can spread invading the visceral pleura. Visceral pleural invasion (VPI) is a form of a locoregional spread of NSCLC and is considered a poor prognostic factor. It is defined as tumor cells invading past the elastic layer (PL1), or to the pleural surface (PL2).^1^, ^2^ VPI in NSCLC is a prognostic factor of poor overall survival (OS) independent of tumor size in stage I patients, it increases risk of recurrence especially in patients with adenocarcinoma,^3^ and pT1 tumors are upstaged to pT2.^4^ In pulmonary adenocarcinoma (PA), invasion of the visceral pleura is reported to be an independent predictor of poor OS and disease‐free survival (DFS) with hazard ratios stronger than tumor size and histologic differentiation.^5^ VPI is associated with more frequent locoregional recurrence and distant metastases.^6^ Another neoplasm that involves the pleura is malignant mesothelioma. It is the most common primary pleural malignant neoplasm that develops from mesothelial cells. Both cancers involving the pleura – adenocarcinoma of the lung and malignant mesothelioma ‐ differ in many clinicopathologic parameters, including OS with patients surviving less than a year for the latter disease.^7^, ^8^

The nucleolus is a prominent structure in the interphase nucleus composed mainly of ribosomal RNA and more than 400 proteins of which nucleolin and nucleophosmin are the most abundant.^9^, ^10^ Nucleolin and nucleophosmin are detected mostly in the nucleolus but also in the nucleoplasm and cytoplasm.^11^, ^12^ They are multifunctional proteins with numerous overlapping, yet nonidentical functions including nucleolus formation, ribosome biogenesis, nuclear‐cytoplasmic protein transport, chromatin remodeling, transcription of rRNA and mRNA, maturation of tRNA, DNA replication and repair, and they function as chaperons and regulators of many cellular factors such as c‐myc and p53.^9^, ^10^, ^12^, ^13^, ^14^ Nucleophosmin functions both as a suppressor and promotor of carcinogenesis,^13^ and it has been shown to be a biomarker of metastatic potential^15^ and acts as an oncoprotein upregulating proliferation, transformation and invasion of cancer cells.^16^ Nucleolin is a pro‐oncogenic protein promoting proliferation and blocking apoptosis^17^ but it can also show the opposite effect by modulating expression of several transcription factors ie, c‐myc.^9^ Altered nucleolin and nucleophosmin expression has been found in many diseases including cancer. Nucleolin expression has been studied in different cancers including breast,^11^ prostate,^18^ colon,^19^ lung,^20^ pancreas,^21^ stomach,^22^ and testicular tumors, ^23^ while nucleophosmin has been studied in a wide variety of solid tumors including cancer of the lung,^16^ bladder,^24^, ^25^ oral cavity,^26^ colon,^27^ liver,^28^, ^29^ thyroid^30^ and in renal tumors.^31^

Only a few studies have focused on the nucleolus in pulmonary tumors. Xu *et al*. found nucleolar size (inconspicuous vs. prominent) to be associated with lymph node and distant metastases of pulmonary adenocarcinoma.^32^ A study on peritoneal malignant mesothelioma showed significant survival differences in relation to nucleolar size with the shortest survival in cases with the largest nucleoli.^33^ In their study, Habougit *et al*. found that small nucleoli or absent nucleoli were associated with better survival in epithelioid pleural malignant mesothelioma.^8^

VPI of pulmonary adenocarcinoma and pleural infiltration of malignant mesothelioma can be considered examples of locoregional spread of cancer. Recently, we studied the nuclear nucleolin and nucleophosmin expression in prostate cancer and its locoregional spread to seminal vesicles. We found a decreased expression of both proteins studied in seminal vesicles infiltrated by prostate cancer compared to cancer cells confined to the prostate gland.^18^ In the current study, we analyzed the nucleolin and nucleophosmin expression and subpopulations of nucleolin‐ and nucleophosmin‐positive cells in PA with pleural invasion and cells of malignant mesothelioma.

## Methods

### Tissue samples

We collected 19 samples from patients with primary pulmonary adenocarcinoma (PA) which had invaded the pleura and 37 samples of pleural malignant mesotheliomas that were pathologically examined in the Independent Laboratory of Pathology “Zdunomed” LLC between 01 January 2009 and 07 July 2017. A total of 9 patients with pulmonary adenocarcinomas underwent lobectomy while 10 were treated with segmentectomy. Out of 37 cases of malignant mesothelioma, we selected 29 cases of epithelial subtype for further study. There were eight cases comprised of six samples of biphasic, one case of desmoplastic and one case of sarcomatoid malignant mesothelioma that were considered not representative enough to include in the study group. In the PA samples, we analyzed the protein expression in the central part of the tumor as well as in pleural invasion. Tissue samples for routine pathologic examination were formalin‐fixed and paraffin embedded. All cases were reviewed by an experienced pulmonary pathologist (PW) and representative slides and paraffin‐embedded tissue blocks were selected for immunohistochemical studies.

### Immunohistochemistry methods and analysis

**Figure 1:**
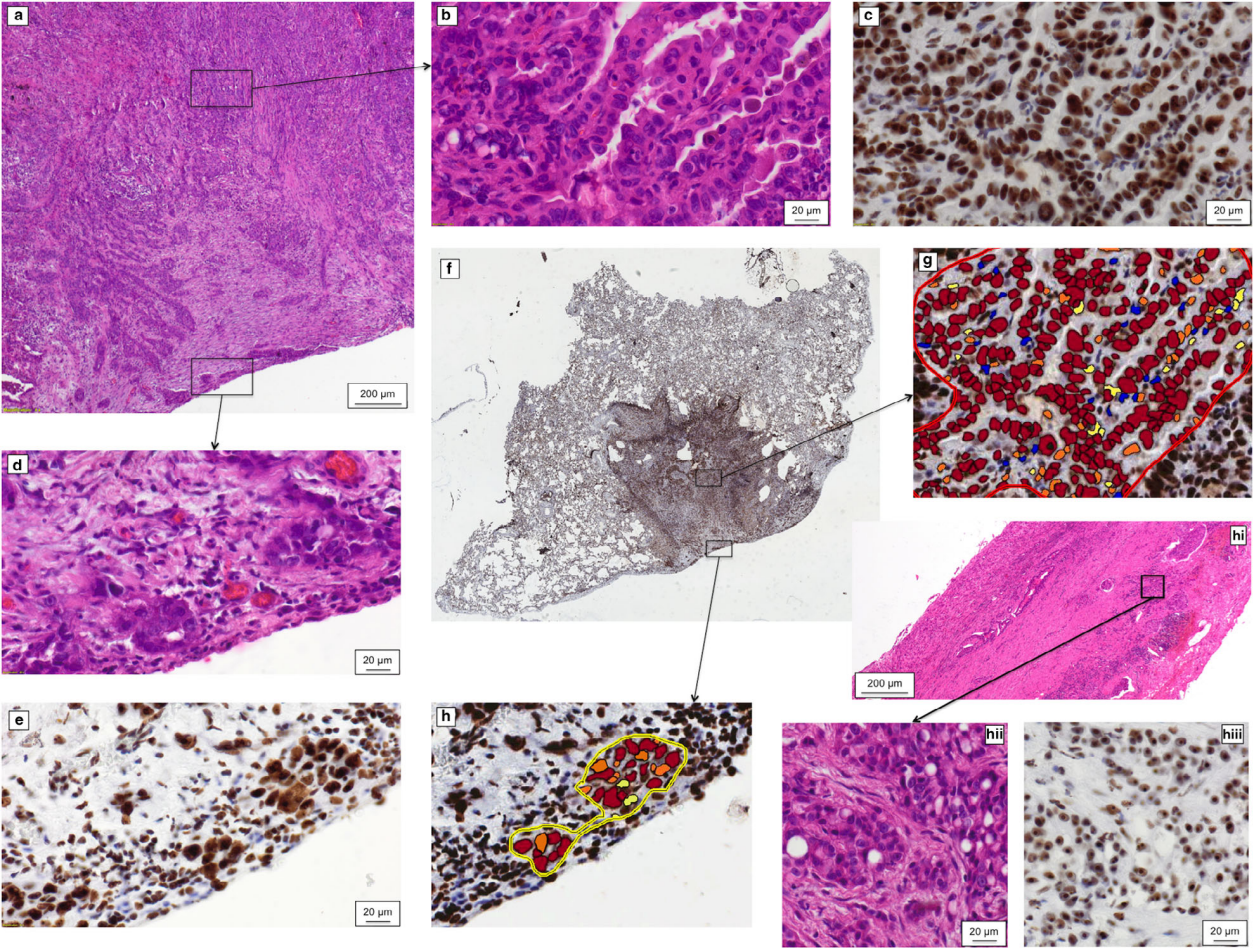
(**a**) Section of pulmonary adenocarcinoma invading the pleura stained with hematoxylin and eosin (HE). (**b**) Higher magnification of the central part of the tumor in pulmonary adenocarcinoma stained with HE. (**c**) Immunohistochemical detection of nucleolin in the central part of the tumor in pulmonary adenocarcinoma. (**d**) Higher magnification of peripheral ‐ pleura invading part of the tumor in pulmonary adenocarcinoma stained with HE. (**e**) Immunohistochemical detection of nucleolin in the peripheral ‐ pleura invading region of pulmonary adenocarcinoma. (**f**,**g**,**h**) Immunohistochemical detection of nucleolin – image analysis. (**f**) Whole section scanned image of pulmonary adenocarcinoma. (**g**) Image analysis of region of interest in the central part of the tumor in pulmonary adenocarcinoma. (**h**) Image analysis of region of interest in peripheral‐ pleura invading region of pulmonary adenocarcinoma. (**hi**) Pleural malignant mesothelioma stained with HE. (**hii**) Higher magnification of pleural malignant mesothelioma (HE). (**hiii**) Immunohistochemical detection of nucleolin in pleural malignant mesothelioma.

Paraffin blocks were cut into 3 μm sections, deparrafinized and antigens retrieved for immunohistochemical reactions. Slides were incubated with primary antibodies after blockage of endogenous peroxidase. For the detection of nucleolar proteins, two mouse antihuman antibodies were used – anti‐nucleophosmin (monoclonal antibody; clone FC82291; 1:2500 dilution; Abcam, Cambridge, UK) and antinucleolin (monoclonal antibody; clone 4E2; 1:2000 dilution; Abcam, Cambridge, UK). Immunohistochemical reactions were visualized with EnVision FLEX/HRP (Dako, Glostrup, Denmark). Slides were counterstained with hematoxylin, dehydrated and sealed with coverslips. Several antigen retrieval procedures were tested and antibodies were titrated for optimal visualization of immunohistochemical reactions. Slides were immediately scanned with ScanScope slide scanner (Aperio Technologies Inc., Vista, CA, USA) and the results of immunohistochemical reactions were analyzed with Aperio ImageScope software using a nuclear protocol. Regions of interest were drawn manually around nests of cancer cells to avoid other tumor elements such as stroma, necrosis, desmoplasia and inflammatory infiltrations. In pulmonary adenocarcinoma cases, we separately selected regions of interest around cancer cells invading the pleura and around cancer cells in the central part of the tumor. Fig 1 shows an example of the hematoxylin and eosin stained slides of pulmonary adenocarcinoma and pleural malignant mesothelioma, as well as images of immunohistochemical reactions and image analysis for nucleolin. The nuclear expression of proteins was analyzed as mean of percentages of all positive cells, percentages of cells in three groups of intensity of reaction: +3, +2, +1 as well as of H‐score.

H‐score was calculated with the formula as follows:

$$H-\text{score}=\left(\%\text{of}+3-\text{positive cells}\times 3\right)+\left(\%\text{of}+2-\text{positive cells}\times 2\right)+\left(\%\text{of}+1-\text{positive cells}\right).$$

The study was approved by the Bioethical Commission of the Pomeranian Medical University in Szczecin, Poland (approval number KB‐0012/268/09/18).

### Statistical analysis

The data distribution was tested with the Shapiro‐Wilk's test and nonparametrical tests were used for determining the differences between groups. A *P*‐value less than 0.05 indicated statistical significance. All analyses were performed with Statistica for Windows 13.1 (StatSoft Inc., Krakow, Poland).

## Results

Mean age of patients with pulmonary adenocarcinoma was 61.8 ± 7.9 year, while patients with malignant mesothelioma was 64.7 ± 8.8. Mean adenocarcinoma tumor size was 38.17 mm ± 16.21 mm (mean ± SD). Lymph node (LN) status of adenocarcinoma cases was four cases of LN+, 10 cases of LN(−), and four LN cases of unknown status. The mean number of cells analyzed for nucleolin expression in the central part of the tumor in pulmonary adenocarcinoma was 6176 ± 1045, in pleural infiltration of pulmonary adenocarcinoma was 6186 ± 1470 and in malignant mesothelioma was 7832 ± 2645 (mean ± SD), while for nucleophosmin expression the number of cells analyzed was 6252 ± 1162, 5574 ± 1005 and 8573 ± 1303, respectively.

### Nuclear nucleolin and nucleophosmin expression in pulmonary adenocarcinoma

We analyzed the nuclear nucleolin expression in primary pulmonary adenocarcinoma in the central part of the tumor and in the cancer cells which were invading the pleura. We found a lower nucleolin expression indicated by an H‐score, as well as a lower percentage of all nucleolin‐positive cells and a lower percentage of highly (+3)‐positive cells in cancer cells invading the pleura. We also found a higher percentage of nucleolin‐negative cells invading the pleura than in the central part of tumors (Table 1).

**Table 1:** Nucleolin expression in the central part of tumors in pulmonary adenocarcinoma and pleural infiltration

	Pulmonary adenocarcinoma – central part of tumors (mean ± standard deviation)	Pulmonary adenocarcinoma – pleural infiltration (mean ± standard deviation)	*P*‐value
% of positive nuclei	91.53 ± 4.89	85.07 ± 6.61	0.01
% of 3+ positive nuclei	63.85 ± 13.02	50.90 ± 14.17	0.02
% of 2+ positive nuclei	19.58 ± 5.87	22.76 ± 6.37	0.29
% of 1+ positive nuclei	8.09 ± 3.74	11.34 ± 4.15	0.06
% of negative nuclei	8.47 ± 4.88	13.82 ± 7.02	0.04
H‐score of nucleolin expression	238.69 ± 25.74	209.57 ± 29.51	0.02

The analysis of nucleophosmin expression showed higher nucleophosmin expression (H‐score) in cancer cells invading the pleura than in central parts of tumors. We also found differences in percentages of subpopulations of cells showing different levels of nucleophosmin expression. The percentage of cells with high (+3) nucleophosmin expression was higher in cancer cells invading the pleura while percentages of cells with intermediate (+2) and low (+1) expression were higher in the central parts of tumors (Table 2).

**Table 2:** Nucleophosmin expression in the central part of tumors in pulmonary adenocarcinoma and pleural infiltration

	Pulmonary adenocarcinoma – central part of tumors (mean ± standard deviation)	Pulmonary adenocarcinoma – pleural infiltration (mean ± standard deviation)	*P*‐value
% of positive nuclei	92.16 ± 4.29	95.17 ± 2.24	0.05
% of 3+ positive nuclei	62.16 ± 6.60	73.00 ± 7.54	0.002
% of 2+ positive nuclei	21.82 ± 6.32	16.44 ± 4.77	0.02
% of 1+ positive nuclei	8.19 ± 1.78	5.73 ± 2.00	0.002
% of negative nuclei	7.83 ± 14.94	4.83 ± 2.23	0.05
H‐score of nucleophosmin expression	237.25 ± 14.12	257.61 ± 12.94	0.01

### Nuclear nucleolin and nucleophosmin expression in pulmonary adenocarcinoma and malignant mesothelioma

We compared nucleolin and nucleophosmin expression in two pleura‐invading neoplasms: in pulmonary adenocarcinoma cells invading the pleura and in malignant mesothelioma. We found significantly higher nucleolin expression in pulmonary adenocarcinoma than in malignant mesothelioma as a percentage of all nucleolin‐positive cells, percentage of highly (3+)‐positive cells and as an H‐score. The percentage of low (+1)‐positive and nucleolin‐negative cells was higher in malignant mesothelioma than in pulmonary adenocarcinoma (Table 3).

**Table 3:** Nucleolin expression in pulmonary adenocarcinoma infiltrating the pleura and malignant mesothelioma

	Pulmonary adenocarcinoma – pleural infiltration (mean ± standard deviation)	Malignant mesothelioma (mean ± standard deviation)	*P*‐value
% of positive nuclei	85.07 ± 6.61	76.28 ± 13.48	0.03
% of 3+ positive nuclei	50.90 ± 14.17	35.18 ± 20.43	0.01
% of 2+ positive nuclei	22.76 ± 6.37	25.19 ± 6.65	0.25
% of 1+ positive nuclei	11.34 ± 4.15	15.91 ± 6.57	0.02
% of negative nuclei	13.82 ± 7.02	23.72 ± 13.47	0.01
H‐score of nucleolin expression	209.57 ± 29.51	171.84 ± 51.62	0.01

The nucleophosmin expression did not show any significant differences between pulmonary adenocarcinoma invading the pleura and malignant mesothelioma (data not shown).

## Discussion

To our knowledge, this is the first analysis of nucleolin and nucleophosmin expression in pulmonary adenocarcinoma invading the pleura and in malignant mesothelioma. We also studied the differences in nuclear nucleolin and nucleophosmin expression in the central part of the tumor in pulmonary adenocarcinoma and in the cancer cells invading the pleura.

We found a lower nucleolin expression, lower percentage of all nucleolin‐positive cells and lower percentage of highly (+3)‐positive cells, as well as a higher percentage of nucleolin‐negative cells in pulmonary adenocarcinoma cancer cells invading the pleura than in the central part of the tumor. Results of our previous study on prostate cancer showed that locoregional spread of prostate cancer (from prostate gland to seminal vesicles) was related to a decrease in nucleolin expression.^18^ Wu *et al*. analyzed 30 cases of colon cancer as primary tumor, lymph node metastases and distal (liver) metastases. They found a gradual loss of nuclear nucleolin expression from primary tumor through lymph node to distant metastases.^19^ Xu *et al*. also studied nucleolin expression in 225 cases of NSCLC and reported that nuclear nucleolin was lower in higher T stages; however, there were no differences between nucleolin status (low vs. high) and histological type. Longer overall survival and disease‐free survival were correlated with higher nucleolin expression and better overall survival was observed independently for the adenocarcinoma group.^20^ The results in our current study are similar to the results of the above‐mentioned studies. Lower nucleolin expression and a higher percentage of nucleolin‐negative cells in cancer cells invading the pleura indicate involvement of this protein in locoregional spread of pulmonary adenocarcinoma. However, we also showed that a subpopulation of cells with lower nucleolin expression might be more invasive than those cells highly expressing nucleolin.

Lower nucleolin expression was reported to be correlated with shorter overall survival of patients with stage II pancreatic ductal adenocarcinoma, ^21^ while in gastric cancer the overexpression of nucleolar nucleolin was found to be the only independent prognostic factor correlated with better prognosis (longer overall survival).^22^

The analysis of nucleophosmin expression in pulmonary adenocarcinomas with pleural invasion in the current study showed a higher nucleophosmin expression and higher percentage of cells with high (+3) nucleophosmin expression while lower percentages of cells with intermediate (+2) and low (+1) nucleophosmin expression in cancer cells invading pleura than in the central part of tumors. This may indicate that pulmonary adenocarcinoma cells with higher nucleophosmin expression are more prone to locoregional spread. A study by Kim *et al*. on several NSCLC cell lines has shown that overexpression of nucleophosmin stimulated colony formation while depletion of nucleophosmin reduced cell growth. Nucleophosmin acted as an oncoprotein upregulating proliferation, transformation and invasion in A549 cells. Depletion of nucleophosmin increased G1 suggesting that loss of this protein imposes a block before S‐phase entry, while nucleophosmin overexpression increased cell population in S phase. Depletion of nucleophosmin made A549 cells highly sensitive to DNA damage‐induced apoptosis, while nucleophosmin overexpression decreased irradiation‐induced cell death. Thus nucleophosmin is also considered an antiapoptotic protein.^16^ In 132 cases of bladder cancer studied by Tsui *et al*. the nucleophosmin expression (low vs. high) correlated with histologic grade, tumor stage and recurrence. The higher percentage of cells with high nucleophosmin expression was found in higher histological grades of cancer, higher tumor stages and recurrent tumors.^25^ Nucleophosmin overexpression in bladder cancer was also found at mRNA level and correlated with higher tumor stage and tumor recurrence. The authors concluded that nucleophosmin overexpression was associated with bladder cancer recurrence and its progression to higher stages.^24^ Increased nucleophosmin expression in oral squamous carcinoma has been reported to be associated with local recurrence and shorter disease‐free survival; however, overall survival was not different in nucleophosmin‐low and nucleophosmin‐high cases.^26^ Liu *et al*. has shown that high nucleophosmin expression correlates with lymph node metastases of colon cancer. The authors concluded that nucleophosmin plays a role in enhanced invasiveness of cancer. Downregulation of nucleophosmin inhibited cancer cell proliferation and impaired migration and invasiveness of colon cancer cell lines while exogenous nucleophosmin expression enhanced cell migration and invasion.^27^ The results of migration and invasion assays of the HCT116 colon cancer cell line are consistent with the results of our current study showing higher nucleophosmin expression in lung adenocarcinoma cells invading the pleura. Nucleophosmin expression has been reported to be associated with cancer cell differentiation. In hepatocellular carcinoma, expression level was determined to be positively correlated with histological grade,^28^ while Xu *et al*. has shown that HMBA‐induced differentiation of liver cancer cells resulted in a decrease in nucleophosmin expression on protein and mRNA levels.^29^ These results may indicate that nucleophosmin may be involved in tumor progression.

This is the first study dedicated to the analysis of nucleolin and nucleophosmin expression in pleura‐invading adenocarcinoma of the lung and pleural malignant mesothelioma. Cells of malignant mesothelioma showed lower nucleolin expression compared to adenocarcinoma cells invading the pleura. The percentages of low (+1) nucleolin‐positive and nucleolin‐negative cells were higher in malignant mesothelioma. This suggests that lower nucleolin expression is correlated with the aggressiveness of neoplasms; however, these results should be considered with caution and further studies are required to explain the role of nucleolin in these two neoplasms. The results of our previous study on nucleolin in testicular tumors also showed lower nucleolin expression in more aggressive nonseminomatous tumors than in less aggressive seminomas.^23^

The nucleophosmin expression did not show any significant differences between pulmonary adenocarcinoma invading the pleura and malignant mesothelioma. The published results of studies on nucleophosmin expression in different tumor types of one anatomic location seem ambiguous. Pianta *et al*. analyzed nucleophosmin expression in thyroid tumors and found differences in nucleophosmin expression between benign (follicular adenomas) and malignant tumors (papillary carcinoma, follicular carcinoma and undifferentiaded carcinoma); however, expression in benign follicular adenoma was higher than in highly aggressive undifferentiated carcinoma.^30^ The results of the study by Sari *et al*. on some histologic types of renal cell carcinomas and oncocytoma showed that nucleolar nucleophosmin expression was higher in benign oncocytoma and highly aggressive sarcomatoid carcinoma than in clear cell renal cancer or chromophobe cancer. The authors also found a correlation between nucleolar nucleophosmin staining and tumor grade.^31^ The results from the above‐mentioned studies suggest that nucleophosmin expression is highly tumor specific. The lack of differences in nucleophosmin expression between adenocarcinoma and malignant mesothelioma that we found in our study are difficult to explain, but may indicate that this protein is similarly involved in the growth of these two neoplasms.

## Disclosure

The authors declare that they have no conflicts of interest.
